# Molecular Pathogenesis of Nonalcoholic Steatohepatitis- (NASH-) Related Hepatocellular Carcinoma

**DOI:** 10.1155/2018/8543763

**Published:** 2018-08-29

**Authors:** Ozlem Kutlu, Humeyra Nur Kaleli, Ebru Ozer

**Affiliations:** ^1^Nanotechnology Research and Application Center, Sabanci University, Istanbul, Turkey; ^2^Center of Excellence for Functional Surfaces and Interfaces for Nano Diagnostics (EFSUN), Sabanci University, Istanbul, Turkey; ^3^Molecular Biology, Genetics and Bioengineering Program, Faculty of Engineering and Natural Sciences, Sabanci University, Istanbul, Turkey

## Abstract

The proportion of obese or diabetic population has been anticipated to increase in the upcoming decades, which rises the prevalence of nonalcoholic fatty liver disease (NAFLD) and its progression to nonalcoholic steatohepatitis (NASH). Recent evidence indicates that NASH is the main cause of chronic liver diseases and it is an important risk factor for development of hepatocellular carcinoma (HCC). Although the literature addressing NASH-HCC is growing rapidly, limited data is available about the etiology of NASH-related HCC. Experimental studies on the molecular mechanism of HCC development in NASH reveal that the carcinogenesis is relevant to complex changes in signaling pathways that mediate cell proliferation and energy metabolism. Genetic or epigenetic modifications and alterations in metabolic, immunologic, and endocrine pathways have been shown to be closely related to inflammation, liver injury, and fibrosis in NASH along with its subsequent progression to HCC. In this review, we provide an overview on the current knowledge of NASH-related HCC development and emphasize molecular signaling pathways regarding their mechanism of action in NASH-derived HCC.

## 1. Introduction

**Figure 1:**
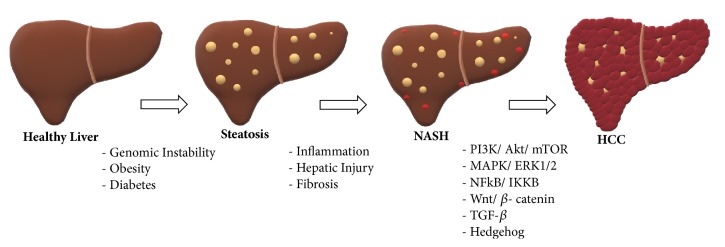
Development of NASH and HCC from healthy liver.

Hepatocellular carcinoma (HCC) is an aggressive cancer with poor prognosis and its incidence increases exponentially in developing countries. The most common underlying causes of HCC are chronic liver diseases and cirrhosis, largely occurring due to hepatitis B, hepatitis C virus (HCV), or alcoholic liver disease [1]. In recent years, nonalcoholic fatty liver disease (NAFLD) also becomes one of leading etiologies for HCC. NAFLD is a spectrum of liver diseases ranging from simple steatosis to liver injury. The initial stage of an inflammatory phase in NAFLD is defined as nonalcoholic steatohepatitis (NASH) [2]. NASH is characterized by inflammation, hepatocellular damage, and fibrosis, which increase the risk of HCC with high rates of mortality (Figure 1). The emergence of HCC in NASH patients with or without cirrhosis is still controversial, such that HCC can also be seen in NASH patients without cirrhosis [3].

**Figure 2:**
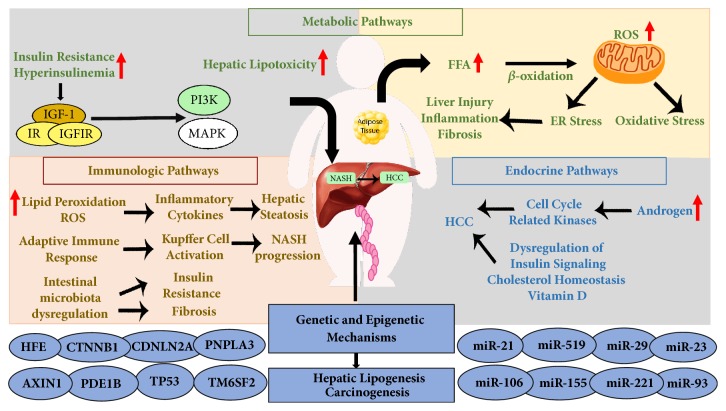
The proposed mechanisms in NASH-related HCC progression.

The progression of NASH-related HCC is a complex and multifactorial process, including several risk factors such as genomic instability, obesity, or diabetes [4, 5]. Involvement of the mechanisms related to these factors appears to cause changes in some common signaling pathways that lead to transition of dysplastic hepatocytes into hepatocellular carcinoma. Currently, the proposed mechanisms include genetic, metabolic, immunologic, and endocrine pathways, which subsequently activate oncogenic mechanisms [6] (Figure 2). In this review, we attempt to summarize recent knowledge in NASH progression and particularly focus on molecular signaling pathways involved in the conversion of NASH into hepatocarcinogenesis.

## 2. Cellular Mechanisms in NASH Progression

Clinical and epidemiological studies support a concept that multiple mechanisms derive NAFLD, NASH, and HCC development. So far, the detailed mechanism of the progression from NAFLD to NASH has not been completely explained, yet a “two-hit hypothesis” was initially proposed [7, 8]. According to this hypothesis, the first hit was insulin resistance, and steatosis was the initiative cause of NASH progression [9]. Insulin resistance enhances lipolysis and increases the level of serum free fatty acid (FFA). Elevation of FFA leads to delivering triglycerides from the liver to peripheral organs, which induce hyper-synthesis of lipid thus causing excessive lipid storage in the liver, called steatosis. Meanwhile, accumulation of triglycerides promotes the appearance of the second hit, oxidative stress, that shows steatohepatitis because of increased level of fatty acid oxidation [10]. Oxidative stress triggers lipid peroxidation, release of proinflammatory molecules, and mitochondrial damage [11], which are the cellular mechanisms involved in the formation of hepatocellular damage, inflammation, and fibrosis in NASH pathology [12, 13].

**Figure 3:**
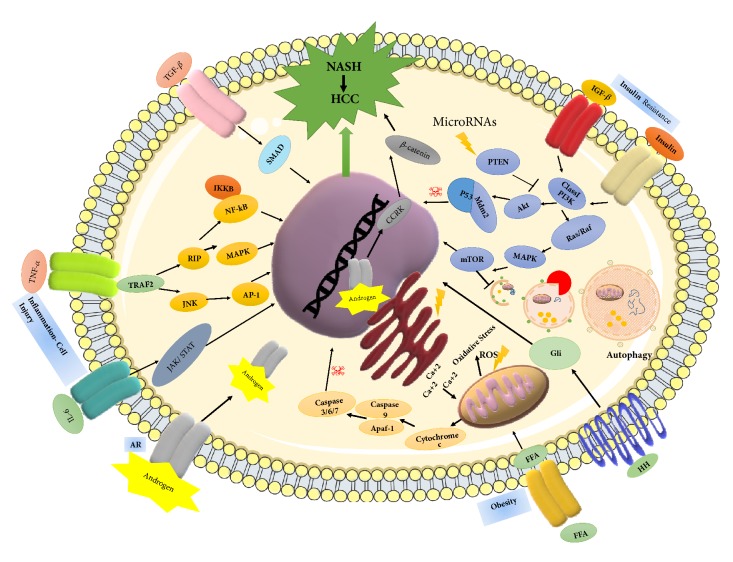
Molecular signaling pathways involved in NASH-related HCC.

**Figure 4:**
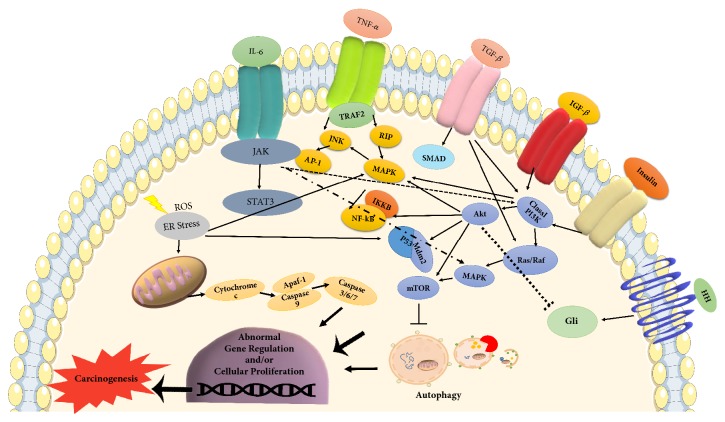
Interaction of oncogenic pathways in NASH-HCC progression.

Even though a large number of researches have supported the two-hit hypothesis, it is still unclear whether NASH develops sequentially on the background of a fatty liver, or it is rather a de novo response to the accumulated lipotoxicity. Instead of the two-hit hypothesis, there is a new consensus on the multiparallel hit theory, which better explains NASH development and its progression to HCC [14]. This theory suggests that NASH is the consequence of numerous conditions acting in parallel, including genetic variations, abnormal lipid metabolism, oxidative and/or endoplasmic reticulum stress, mitochondrial dysfunction, altered immune responses, and imbalance in gut microbiota [15]. According to this theory, hepatic inflammation is the first cause of fibrosis progression in NASH rather than steatosis [16]. The following section will focus on detailed mechanisms at molecular level and their related signaling pathways in NASH-dependent HCC progression (Figures 3 and 4).

## 3. Molecular Mechanisms Involved in NASH-Related HCC

### 3.1. Genetic and Epigenetic Mechanisms

Recent advances in genetic technology allow obtaining comprehensive data on the genetic alterations associated with HCC. Differential gene expression results from gene mutations in regulatory elements or epigenetic changes, which plays an important role in susceptibility to the development of HCC.

Genetic mutation in the gene encoding patatin-like phospholipase domain-containing protein 3 (PNPLA3) on chromosome 22 is a well-known factor in NASH-related HCC progression [17]. The variant (rs738409 c.444 C>G, p.I148M) causes a cytosine to guanine mutation resulting in isoleucine to methionine conversion. This mutation correlates with increased lipid accumulation in liver and predisposes individuals to fatty liver-associated diseases, from simple steatosis to steatohepatitis, NASH, and HCC [18]. Although the physiological and biological functions of PNPLA3 within the liver are not fully elucidated, the association of PNPLA3 mutations with HCC is evident [19]. Overexpression of I148M variant in mouse liver promotes accumulation of triacylglycerol, increases synthesis of fatty acids, and impairs triacylglycerol hydrolysis [20]. Moreover, the PNPLA3 genotype has been reported to influence liver storage of retinol and retinol serum levels in obese subjects [21] suggesting a potential role of PNPLA3 in regulating retinol metabolism and hepatic stellate cells (HSCs) biology. Similarly, PNPLA3 has been shown to be expressed in HSCs [22], but its role in HCC progression in these cells still needs to be investigated [23]. There is an increased prevalence of another mutation in the transmembrane 6 superfamily member 2 gene (TM6SF2) in NASH patients. Carriage of a genetic variant in TM6SF2 (rs58542926 c.449 C>T, p.E167K) on chromosome 19 (19p13.11) has been reported to correlate with steatosis and advanced fibrosis in NASH patients [24, 25], independently of diabetes, obesity, or PNPLA3 genotype. Although, conflicting data exists regarding its role in HCC progression, the TM6SF2 variant is thought to be associated with liver injury in NASH-related HCC pathogenesis [26]. Hemochromatosis gene (HFE) mutations (C282Y and H63D) in NASH increased the susceptibility to more severe form of disease with fibrosis or cirrhosis [27, 28] and implicated HCC development in these patients. Particularly, H63D mutation was found in noncirrhotic HCC and led to hepatic inflammation, fibrosis, and carcinogenesis due to increased iron load in these patients [29]. Recently, the rs641738 genotype, encoding the membrane bound O-acyltransferase domain-containing 7 (MBOAT7), was associated with more severe liver damage and increased risk of fibrosis in NASH patients; however, these findings need further investigation regarding HCC progression [30, 31]. In addition to various single mutations, the genetic instability in NASH patients was reported much higher than in NAFLD patients, and this was considered as one of the inducements for NASH-related HCC. Quantitative analysis revealed abundant amplifications of DNA, where the genes involved in oncogenic mechanisms are located. These genes encode telomerase reverse transcriptase (TERT), vascular endothelial growth factor A (VGFA), MET, and MYC proteins that are known to have a role in tumor growth. Moreover, exome-sequencing analysis of HCC showed the highest prevalence of mutation in oncogenic genes, like CTNNB1, AXIN1 (involved in *β*-catenin/WNT signaling pathway), albumin (ALB), TP53, and CDKN2A [32]. Furthermore, differential expressions of the exportin 4 (XPO4) and phosphodiesterase 1B (PDE1B) genes were identified in HCC as well as in NASH; however, the physiological role of these genes in NASH-related HCC is still unknown [33, 34].

Epigenetic changes, causing aberrant DNA methylation, have been considered another important mechanism in NASH progression [35]. It occurs through the enzyme methyltransferases (DNMTs), leading to silence of genes related to DNA damage and repair, lipid and glucose metabolism, and fibrosis progression [36]. The methylation of the CpG island near the PDE1B gene was shown to be linked with survival in HCC patients; nevertheless, the only epigenetic change that has clearly been linked to NASH-related HCC is the gene encoding chromodomain helicase DNA-binding protein 1 (CHD1) [37].

MicroRNAs (miRNAs) are endogenous, small noncoding RNAs, having a role in the regulation of gene expression. Convincing evidence showed that expression of miRNAs is dysregulated in many cancers through various mechanisms and they may function as either oncogenes or tumor suppressors under certain conditions [38]. So far, no studies have yet significantly focused on miRNA expression in human NASH-associated HCC; however, genome-wide analysis revealed 23 miRNAs, differentially expressed in NASH patients. Among them, liver specific miR-122 expression is reduced in NASH patients and, thus, negatively regulates hepatic lipogenesis [39]. Downregulation of miR-122 was also demonstrated in a mouse model of NASH-HCC, indicating direct role of this miRNA in NASH-associated HCC [40]. To date, most of the studies indicate a critical role of several miRNAs (miR-21, miR-29, miR-23, miR-155, miR-221, miR-222, miR-106, miR-93, miR-519) in NASH-associated carcinogenesis [32]. Strikingly, altered expression of these miRNAs have been found to be involved in major hepatocarcinogenic pathways, including the TGF-*β*, Wnt/*β*-catenin, mitogen-activate protein kinase (MAPK), and phosphatidylinositol 3-kinases (PI3K)/AKT/mTOR that regulate proliferation and energy metabolism in the cell [41]. Importantly, several of these miRNAs target the main inhibitor of the PI3K/AKT pathway, PTEN protein, and its mutations were found in HCC patients [42]. In accordance, PTEN deficient mice have been shown to develop steatosis, hepatomegaly, and HCCs [43, 44].

### 3.2. Metabolic Pathways

The common association of high-fat diet, obesity, and diabetes with NASH and HCC pathogenesis indicates that the molecular link between energy balance and cell cycle control in hepatocytes is the key mechanism for the progression of NASH-related HCC. Indeed, these metabolic factors are closely related to insulin resistance and hyperinsulinemia, which activates insulin receptor signaling via PI3K and MAPK pathway.

Experimental evidence indicated that insulin resistance and hyperinsulinemia increased the expression of insulin and insulin-like growth factor-1 (IGF-1) [45]. Binding of insulin or IGF-1 to their respective receptors, namely, insulin receptor (IR) and insulin-like growth factor-1 receptor (IGF1R), triggers signaling cascade via insulin receptor substrate-1 (IRS-1) that results in activation of its downstream PI3K and MAPK pathways. In fact, these pathways play a significant role in the carcinogenesis of HCC by induction of cell proliferation and inhibition of apoptosis [46, 47]. The role of PI3K pathway in the progression of HCC is mainly mediated by its effect on cyclin D1-dependent cell cycle, Mdm2/p53-dependent apoptosis, and mTOR-dependent cell growth [48]. On the other hand, MAPK pathway affects cell growth by inducing the transcription of protooncogenes, c-fos, and c-jun. In addition, MAPK pathway eventually activates the Wnt/*β*-catenin signaling cascade, which leads to fibrosis and carcinogenesis in liver [49].

Another important consequence of insulin resistance is excessive lipid accumulation in liver. In other words, imbalance in energy metabolism increases hepatic lipotoxicity, resulting in excessive production of FFAs [50]. Indeed, *β*-oxidation of these FFAs in mitochondria induces the formation of reactive oxygen species (ROS). Overproduction of ROS causes respiratory chain disruption and further functional defect in mitochondria, which is the main event for cytochrome c release and triggering apoptotic death signal. Recently, RIP1- and RIP3-activated JNK (Jun-(N)-terminal kinase) has been proposed as an apoptotic pathway responsible for the emergence of liver injury, inflammation, and fibrosis in NASH patients as well as in mouse model of steatohepatitis [51, 52].

Insulin signaling and lipotoxicity in mitochondria are connected to several other mechanisms, such as oxidative and endoplasmic reticulum (ER) stress, that contribute to hepatic cell injury and ultimately carcinogenesis in NASH [53]. Certainly, there is significant cross-talk between ROS production, oxidative and/or ER stress, and cell death mechanisms, correlating to the development of progressive disease conditions in NASH and HCC. ROS and oxidative stress disrupt ER functions via increased release of calcium from ER stores. Excess amount of calcium level induces mitochondrial and lysosomal permeabilization, which in turn increased further mitochondrial ROS release and potentiate sequential activation of proapoptotic pathway initiated by executive caspases 9 and 3 [54, 55]. Under normal catabolic condition in cells, the superoxides (incompletely reduced forms of oxygen) are converted into nontoxic water by glutathione peroxidase and catalase. The biochemical function of these enzymes is to protect the organism from oxidative damage by reducing the amount of free hydrogen peroxide. The level of iron is an important factor for glutathione peroxidase and catalase activities, which is upregulated during intake of excess iron; otherwise, it induces oxidative stress by enhancing FA oxidation. Accordingly, elevated level of iron is observed in NASH patients and considered as a risk factor for HCC development [56].

Autophagy is one of the important stress response pathways in cells, supporting cell survival by recycling metabolic components. This mechanism reduces cytosolic organelles or macromolecules by sequestering them in double-membrane vesicles and delivering them to the lysosomes for degradation. Recent discoveries showed a molecular connection between lipolysis and autophagy mechanisms. In the liver, autophagy suppresses protein aggregate, lipid accumulation, oxidative stress, chronic cell death, and inflammation. On the contrary, autophagy regulates adipogenesis and adipose tissue differentiation [57]. Now, the emerging role of autophagy in NASH and NASH-derived HCC is a double-edged sword. On the one hand, autophagy enables the hepatocytes to tolerate stress and promote tumorigenesis. On the other hand, autophagy plays an important role in damage mitigation in response to stress that can limit tumorigenesis [58, 59]. Although there is controversy whether autophagy promotes or inhibits NASH progression, its role in energy metabolism via PI3K/mTOR pathway strongly supports the idea that autophagy may be an ideal candidate for therapeutic purposes. Therefore, further investigations are needed to determine the exact role of autophagy in NASH-associated HCC.

### 3.3. Immunologic Pathways

Mitochondrial dysfunction and stimulation of stress mediators not only facilitate the production of ROS, but also contribute to the progression of HCC by immune reactions. Insulin resistance and oxidative stress stimulate IKK*β*- (inhibitor of kappa light polypeptide gene enhancer in B-cells, kinase beta) dependent NF-*κ*B (nuclear factor kappa-light-chain-enhancer of activated B-cells) signaling pathway and promote hepatocyte survival in addition to their crucial role in liver inflammatory responses [60]. It has been shown that ROS along with products of lipid peroxidation increases the release of several inflammatory and inhibitory cytokines such as tumor necrosis factor-alpha (TNF-*α*), interleukin-6 (IL-6), leptin, and adiponectin [61]. TNF-*α* activates prooncogenic pathways via JNK and IKK*β* that promote the synthesis of AP-1 and NF-*κ*B. Phosphorylation and subsequent degradation of IKK*β* lead to the nuclear entry of NF-*κ*B, triggering inflammatory cascades, which in turn aggravate NF-*κ*B activation. Extracellular lipid can also activate IKK*β* by engaging TLRs (Toll-like receptors). The TLR-deficient mice studies revealed the attenuation of severe steatosis, indicating TLR as an important proinflammatory mediator in NASH progression [62]. On the other hand, IL-6 activates STAT-3 (signal transducer and activator of transcription 3), an oncogenic transcription factor that induces cell proliferation and antiapoptotic pathways, and was found to be important for NASH-related HCC development [63]. Leptin has been described as profibrotic and proangiogenic factor in liver carcinogenesis by initiating an intracellular signaling cascade of proinflammatory cytokines (TNF-*α* and IL-6). Moreover, binding of leptin to its respective receptor in HCC cells activates JAK2/STAT, MAPK, and PI3K signaling pathways [64]. Interestingly, leptin has also been shown to upregulate the TERT and thereby lead to immortalization of tumor cells in HCC [65]. Adiponectin is an anti-inflammatory cytokine, specifically produced in adipose tissue. Under normal physiological conditions, it inhibits angiogenesis via modulation of apoptosis [66]. However, insulin resistance reduced level of adiponectin and the release of TNF-*α* and IL-6 that further inhibit adiponectin production and thus potentiate HCC development [67]. Adiponectin and leptin act antagonistically on liver fibrogenesis and inflammation [68]. However, reports of serum levels of adiponectin and the expression of its receptor are inconsistent [69]. Therefore, further investigations are necessary to clarify the function of adipokines in NASH and HCC development.

Immune activation is a prerequisite for the development of NASH, which is also linked to adaptive immune responses. In several animal models, the potential role of CD8+ T-lymphocytes, and CD4+ T-lymphocytes in liver damage and carcinogenesis was demonstrated [70, 71]. Moreover, liver damage stimulates the recruitment of different types of immune cells to the site of injury. Kupffer cell (KC) activation is critical in NASH and precedes the recruitment of other cells, therefore contributing to NASH progression [72]. In NASH, a number of ligands and cytokines can also activate Natural Killer (NK) cells; however, data obtained from animal models are contradictory, indicating that two different phenotypes of NK cells have been associated with liver disease and act oppositely during inflammation [73, 74]. The involvement of adaptive immune system was demonstrated in response to liver injury and inflammation, but its exact role in NASH-related HCC is still unknown.

Acute cell injury triggers another signaling pathway, Hedgehog, a complex cellular pathway for liver repair and regeneration. This pathway induces mobilization of hepatic progenitor cells at the site of injury and replaces damaged hepatocytes [75]. Current data suggest that abnormal Hedgehog signaling results in dysregulated cellular repair and malignant transformation in HCC progression. Moreover, the development of HCC has been described as a contrary function of Hedgehog pathway, in which hyperactivation of progenitor cells could survive independently from regulation of NF-*κ*B, thereby being less susceptible to NF-*κ*B-driven apoptosis [76].

The gut microflora plays an important role in the development and function of the host immune system. Through the portal circulation, liver is directly exposed to gut-derived products, being the first line of defense against bacterial toxins [77]. The studies in both animal models and human showed that alteration in intestinal microflora triggers an immune response, inflammation, and immune cell infiltration of liver and adipose tissue. Modulation of gut microbiota induces insulin resistance by inhibiting expression of gut-secreted anorectic hormones, such as GLP-1 and PYY. In addition, the reduced expression of a LPL- (lipoprotein lipase-) suppressor FIAF (fasting-induced adipose factor) prevents FA release leading to FA and triglyceride accumulation [78]. The shift on the bacterial community prevalence in gut microbiota results in release of pathogen-associated molecular patterns (PAMPs). PAMPs are recognized by TLRs and other pattern recognition receptors (PRRs) and potentiate innate immune responses. Lipopolysaccharides (LPSs), a major component of outer membrane of gram negative bacteria, are considered the prototypical class of PAMPs. While LPSs are specifically recognized by TLR4, the other PAMPs such as flagellin, lipoteichoic acid, peptidoglycan, nucleic acid variants (dsRNA), or unmethylated CpG motifs are recognized by other receptors, such as TLR2, TLR3, TLR5, and TLR9 [79]. Similarly, human TLR2, TLR4, and TLR9 are involved in the pathogenesis of NASH [80]. Interaction of LPSs and TLR4 with the monocyte differentiation antigen CD14 system on Kupffer cells triggers inflammatory cascade, which activates NF-*κ*B pathway and induces the production of TNF-*α*, IL-1, and IL-6 cytokines [81]. The stimulation of this pathway was demonstrated in animal model of NASH, and elevated TNF-*α* expression as well as serum LPS-binding proteins was detected [82]. In HSCs, the activation of TRL4-dependent pathway was shown to be involved in fibrosis progression [83]. Although further investigations are necessary to show the generation of secondary bile acids by gut microbiota in NASH-HCC, the studies have shown the induction of DNA damage by one of the secondary bile acids, sDCA [84, 85].

### 3.4. Endocrine Pathways

The incidence of NASH and HCC is higher in males irrespective of the etiology. This suggests that the differential endocrine signaling might increase the tendency of HCC development in NASH patients. Both estrogen and androgen are steroid hormones that mediate their action by binding to nuclear receptors and acting as transcription factors to regulate the expression of multiple genes. It was suggested that androgen and androgen receptors (ARs) might promote HCC progression and/or that estrogen and estrogen receptors might suppress HCC development [86]. The AR gene encodes AR molecule, which is a transcriptional factor able to bind DNA with its DNA-binding domain. AR is activated directly by androgen hormone and induces the transcription of cell cycle-related kinase (CCRK) that upregulates *β*-catenin/T-cell factor signaling, leading to promotion of HCC [87]. ARs can also be activated by other signaling pathways such as MAPK and PI3K, which are well-known in the development of HCC in NASH [88]. Although ARs are extensively studied in HCC, their role in NASH is still under investigation. Several animal studies demonstrated the development of liver steatosis, insulin resistance, altered lipid metabolism, and progression of NASH to HCC via either SREB1 (sterol regulatory element-binding protein), PEPCK (phosphoenolpyruvate carboxykinase), and PTB-1B (protein tyrosine phosphatase 1B) or SREB2 and CYP27A1 [89]. These molecules play significant role in insulin signaling, cholesterol homeostasis, and vitamin D3 metabolism through activation of the JNK pathway [90].

## 4. Conclusion and Future Perspectives

NASH is the aggressive form of NAFLD and its prevalence is progressively increasing due to the growing epidemic of obesity and diabetes. Accumulated evidence is likely to make NASH one of the most common causes of HCC in upcoming years. Recent advances in whole genome association study (WGAS) and next generation sequencing (NGS) allow clarifying remarkable genetic changes in signaling pathways related to energy metabolism and cell proliferation that are directly linked to carcinogenesis. Currently, the data obtained from various clinical and in vivo molecular studies achieve the consensus that genomic instability, abnormal lipid metabolism, uncontrolled stress mediators, and altered immune responses are coordinately acting mechanisms, prompting inflammation, liver injury, and fibrosis along with HCC. Our understanding of the underlying molecular basis in the NASH-related HCC development is that the signaling pathways involved in NASH pathogenesis seem to act simultaneously in HCC development. In this complex scenario, key molecules involved in reciprocal interaction between several pathways lead to overactivation of prooncogenic mechanism and, meanwhile, inactivate tumor-suppressive or antioncogenic mechanisms. Ongoing clinical trials of a wide range of molecules, targeting different pathways, have been shown to reduce the NASH-HCC progression in several pathogenic aspects, yet the translation of these findings into personalized therapy is still a major challenge. Thus, a better understanding of the molecular signaling pathways involved in NASH-related HCC will allow the discovery of novel targeting molecules for therapeutic and preventive approaches.
